# Patient Characteristics and Outcomes Associated with Sentinel Protection Device Use in Patients with Aortic Valve Disease Undergoing TAVR in a “Real-World” Setting

**DOI:** 10.31083/j.rcm2501003

**Published:** 2024-01-04

**Authors:** Habib Jabagi, Richard E. Shaw, Lara Gharibeh, Rajiv Tayal, Hussein Rahim, Francis Kim, Alex Zapolanski, Juan B. Grau

**Affiliations:** ^1^Division of Cardiothoracic Surgery, The Valley Hospital, Ridgewood, NJ 07450, USA; ^2^Department of Cardiovascular Surgery, Mt. Sinai Hospital, Icahn School of Medicine at Mt. Sinai, New York, NY 10001, USA; ^3^Department of Biochemistry, Microbiology and Immunology, University of Ottawa, Ottawa, ON K1Y 4W7, Canada; ^4^Division of Interventional Cardiology, The Valley Hospital, Ridgewood, NJ 07450, USA; ^5^Division of Cardiac Surgery, University of Ottawa Heart Institute, Ottawa, ON K1Y 4W7, Canada

**Keywords:** sentinel cerebral protection system (CPS), transcatheter aortic valve replacement (TAVR), aortic valve disease, cerebrovascular accidents (CVA), stroke

## Abstract

**Background:**

Transcatheter aortic valve replacement (TAVR) has become the dominant treatment for aortic valve disease. While TAVR safety has improved over time, concern remains over the occurrence of cerebrovascular accidents (CVA) secondary to device placement, which is associated with increased morbidity and mortality. The Sentinel Cerebral Protection System (CPS) was developed to reduce the risk of embolic strokes associated with debris produced during TAVR. Studies evaluating Sentinel CPS efficacy have produced conflicting results, and there is little understanding of which patients are selected for device placement in “real-world” settings. With no existing guidelines on device use, the purpose of this study was to describe and compare the characteristics of patients who receive CPS with those who do not in a “real-world” setting of consecutive TAVR patients and evaluate its impact on postoperative complications, namely stroke.

**Methods:**

This was a single-center, retrospective study of all patients undergoing TAVR between July 1, 2019, and December 31, 2020. Patient demographics, baseline, and perioperative characteristics were collected prospectively using the Society of Thoracic Surgeons (STS)/American College of Cardiology (ACC) Transcatheter Valve Therapy (TVT) Registry and our institution’s TAVR database for analysis. Postoperative outcomes were assessed using primary endpoints of in-hospital/30-day stroke and the composite of death, stroke, and bleeding/vascular events at one-year. To adjust for baseline differences, a propensity score was developed including all factors that were different between groups, and Multivariate Cox Regression analysis was used to control for these differences. Patient follow-up was 97% complete at 12 months with 100% echocardiographic follow-up.

**Results:**

A total of 242 consecutive patients (57.9% male) were analyzed, with a mean age of 79.9 ± 9 years. Of these patients, 134 (55.4%) received the Sentinel CPS and 108 (44.6%) did not. Sentinel CPS patients were more likely to be male, not on dialysis, without prior CVA or pacemaker, had less severe chronic lung disease, and were lower operative risk compared to concurrent non-CPS patients. CPS patients were also found to have higher hemoglobin and albumin levels, lower creatinine, and were less likely to be on immunosuppressant therapy. The incidence of in-hospital/30-day stroke after TAVR did not differ between CPS and non-CPS patients (0.0% vs. 1.9%; *p* = 0.198). Unadjusted analyses at one-year showed a lower occurrence of the composite endpoint in CPS patients compared non-CPS patients (8.3% vs. 17.0%; *p* = 0.034). After adjustment, the hazard ratio (Adj HR) for the CPS group was no longer significantly associated with a lower composite endpoint (Adj HR = 0.609, 95% CI 0.244–1.523; *p* = 0.289). Both unadjusted (*p* = 0.233) and adjusted (*p* = 0.132) analyses showed no difference in the incidence of stroke at one-year.

**Conclusions:**

Our study demonstrates that in a “real-world” setting, the Sentinel CPS device is more likely to be used in healthier and less complex patients. In analyses adjusted for illness severity and patient complexity, CPS use did not have a significant effect on the incidence of in-hospital/30-day stroke or the composite endpoint of death, stroke, and bleeding/vascular events at one-year.

## 1. Introduction

Transcatheter aortic valve replacement (TAVR) has become the most widely used treatment in low to high-risk patients with aortic valve disease, exceeding all other forms of Surgical Aortic Valve Replacement (SAVR) in 2019 [1]. With advances in device technology and increased operator experience, TAVR has become a proven safe and effective treatment method for aortic valve replacement (AVR) [2]. Although these advances have reduced many of the early complications seen with TAVR, such as vascular complications and paravalvular leaks, concern persists over the increased risk of cerebrovascular accidents secondary to embolization of aortic and aortic valve debris during TAVR. Stroke remains an important cause of morbidity and mortality in patients undergoing TAVR [3, 4], in spite of procedure and device evolution [5].

Post-TAVR stroke remains a serious complication associated with increased mortality [6, 7, 8]. The 30-day risk of stroke following TAVR ranges between 1 to 5.5% [3, 9], and one-year stroke rates between 4.3 to 8.2% [8, 9], with the occurrence of stroke associated with a 3.5-fold increase in the risk of death in the first month post-TAVR [3, 5, 10, 11]. Even with the evolution of new TAVR devices, review of the Society of Thoracic Surgeons (STS)/American College of Cardiology (ACC) Transcatheter Valve Therapy (TVT) Registry has shown only slight declines in perioperative and 30-day stroke rates [1], and has further demonstrated stroke risk to be independent of TAVR physician experience [12, 13].

To mitigate TAVR stroke risk, cerebral protection devices were developed to help protect the brain from embolic strokes. These devices deploy temporary filters in the aortic arch and/or great vessels (i.e., brachiocephalic and left common carotid arteries) to capture embolic debris dislodged during TAVR. Among these devices, the Sentinel Cerebral Protection System (Sentinel CPS) is the only Food and Drug Administration (FDA) approved device for use with TAVR in the USA [14, 15], with conflicting evidence surrounding its benefit in stroke reduction [3, 5, 14].

With procedural success rates over 90%, the Sentinel CPS has an excellent safety profile, with low rates of mortality, neurological events, and major adverse cardiac and cerebrovascular events (MACCE) in randomized clinical trials (RCT) [3, 5, 15, 16, 17, 18]. However, the use of this device in real-world settings has not been well-studied, and no guidelines exist on CPS device usage and patient selection in TAVR. Thus, the main objective of this study was to evaluate and compare the clinical characteristics of patients who receive the Sentinel device to concurrent patients who do not in a real-world setting. The primary analysis objective of this study is to evaluate the association of Sentinel CPS use in TAVR on perioperative cerebrovascular accidents (CVA), as well as the occurrence of CVA and a composite outcome of death, CVA, and bleeding/vascular events in consecutive patients undergoing TAVR at 1 year.

## 2. Methods

### 2.1 Study Population

This retrospective study included 242 consecutive patients who presented at a single institution for treatment of aortic valve disease using TAVR between July 1, 2019, and December 31, 2020. Data were collected prospectively using standard elements and definitions from the STS/ACC TVT Registry [19]. The local database was approved by the Western Institutional Review Board (WIRB), and informed consent was waived for this study. Of the 242 TAVR patients, 134 (55.4%) had the Sentinel CPS (Boston Scientific, Marlborough, MA, USA) placed for cerebral protection and the remaining 108 (44.6%) concurrent patients underwent TAVR without Sentinel CPS. During this study period, a total of only 4 patients did not have a Sentinel device placed who were originally scheduled to receive one. These patients were not included in this analysis and were excluded from the study. Sentinel CPS use was commercially funded for use in TAVR by Boston Scientific across the country during the study period.

### 2.2 TAVR Protocol

Each patient underwent TAVR using our institution’s standard protocol. The use of the Sentinel CPS was left to operator choice, taking into consideration anatomical constraints, which may have precluded device deployment, including: aortic arch anomalies, severe arch/great vessel calcification, and right radial artery access. The Sentinel CPS was delivered percutaneously via the right radial artery over a 0.014” guidewire, with the deployment of filters in the brachiocephalic and left common carotid arteries in all patients under fluoroscopy guidance. The filters remained in situ for the duration of the procedure and were removed at the conclusion of the TAVR. Right radial sheaths were removed with the placement of TR BANDs® (Terumo Radial Band, Toyko, Japan), for radial artery compression.

At the time of this study, dual antiplatelet therapy (DAPT) for 6 months after TAVR was the current default strategy as recommended by the most up-to-date 2014 American Heart Association (AHA)/ACC and 2017 European Society of Cardiology (ESC) Guidelines for the Management of Valvular Heart Disease [20, 21]. As such, all patients included in this study were started and maintained on DAPT in accordance with the currently available recommendations. For patients with atrial fibrillation, our center’s strategy was to only resume the patient’s home blood thinner, and not start DAPT following TAVR to reduce bleeding risk [22, 23].

### 2.3 Follow-Up and Endpoints

Patients in this study were followed at 30 days and 12 months post-TAVR, using the STS/ACC TVT Registry follow-up protocol [19]. The primary endpoint at 30-days was the occurrence of CVA (inclusive of in-hospital stroke), and at one-year was a composite endpoint of death, CVA, bleeding, and vascular events. CVA and other follow-up events were defined using standard outcome definitions as defined by The Valve Academic Research Consosroitum-3 (VARC-3) [24]. Both CVA and bleeding/vascular events were diagnosed and confirmed by at least 2 independent physicians (1 Intensivist, 1 Interventional Cardiologist, and 1 Cardiac Surgeon) based on the VARC-3 criteria. Only CVA’s meeting criteria for “permanent stroke” were included in this study. “Permanent stroke” was defined as any confirmed neurologic deficit of abrupt onset caused by a disturbance in cerebral blood supply with duration ≥24 hours, as per STS/ACC TVT registry [19] and STS database [25].

### 2.4 Statistical Analysis 

Continuous data are displayed as means with standard deviation. Categorical data are expressed as proportions. Univariate statistical tests for continuous data included tests of mean differences using the Student’s *t*-test. Categorical variables were analyzed using the Chi-Squared test. Comparison of in-hospital variables were adjusted using either logistic or linear regression with STS Risk score as the adjustment variable. To control for baseline differences between the Sentinel CPS and no Sentinel CPS groups in the analysis of follow-up endpoints, a propensity score was developed using group membership in the treatment group as the dependent variable and all factors listed in Tables 1,2. The propensity score was used as a covariate in the multivariate analyses performed so that the entire consecutive patient experience was preserved. This approach has been compared to the method of using the propensity score to develop matched groups and has been found to produce similar results in adjusting for group differences [26].

**Table 1.** **Baseline clinical characteristics of all TAVR patients and Sentinel CPS vs. no Sentinel CPS groups**.

		All Patients	Sentinel CPS	No Sentinel CPS	*p* value
		N = 242	N = 134 (55.4%)	N = 108 (44.6%)	
Age (mean ± SD)		79.9 ± 9	79.7 ± 9	80.2 ± 8	0.671
BMI (mean ± SD)		29.2 ± 7	28.8 ± 6	29.6 ± 7	0.347
Male		140 (57.9)	88 (65.7)	52 (48.1)	0.006**
Current Smoker		8 (3.3)	5 (3.7)	3 (2.8)	0.485
History of DM		93 (38.4)	46 (34.3)	47 (43.5)	0.092
Current Dialysis		8 (3.3)	1 (0.7)	7 (6.5)	0.016*
History of HTN		220 (90.9)	123 (91.8)	97 (89.8)	0.378
Prior MI		37 (15.3)	17 (12.7)	20 (18.5)	0.142
Heart Failure (within 2 weeks)		218 (90.1)	119 (88.8)	99 (91.7)	0.302
Prior CVA		19 (7.9)	9 (6.7)	10 (9.3)	0.310
Prior TIA		15 (6.2)	4 (3.0)	11 (10.2)	0.020*
History of Carotid Disease		44 (18.2)	20 (14.9)	24 (22.2)	0.446
History of PVD		36 (14.9)	19 (14.2)	17 (15.7)	0.436
Chronic Lung Disease		71 (29.3)	29 (21.6)	42 (38.9)	0.012*
	Mild	49 (202)	17 (12.7)	32 (29.6)	-
	Moderate	9 (3.7)	5 (3.7)	4 (3.7)	-
	Severe	13 (5.4)	7 (5.2)	6 (5.6)	-
History of AF		83 (34.3)	46 (34.3)	37 (34.3)	0.991
Left Main Disease		19 (7.9)	10 (7.5)	9 (8.3)	0.493
Bicuspid Aortic Valve		12 (4.9)	4 (3.0)	8 (7.4)	0.0218
On Immunosuppressant Therapy		12 (5.0)	1 (0.7)	11 (10.2)	<0.001**

Abbreviations: AF, atrial fibrillation; BMI, body mass index; CVA, cerebrovascular accidents; DM, diabetes; HTN, hypertension; MI, myocardial infarction; PVD, peripheral vascular disease; TIA, transient ischemic attack; TAVR, transcatheter aortic valve replacement; CPS, cerebral protection system. Data are presented as n (%), unless otherwise indicated. *p < 0.05, **p < 0.01.

**Table 2.** **Pre TAVR procedural characteristics and patient status**.

		All Patients	Sentinel CPS	No Sentinel CPS	*p* value
		N = 242	N = 134 (55.4%)	N = 108 (44.6%)	
Hemoglobin (mean ± SD)		12.2 ± 1.7	12.5 ± 1.7	11.8 ± 1.7	0.001**
Platelets (mean ± SD)		206,344 ± 74,288	205,053 ± 61,986	207,936 ± 74,288	0.743
Creatinine (mean ± SD)		1.18 ± 0.81	1.07 ± 0.61	1.32 ± 0.98	0.016*
Total Albumin (mean ± SD)		3.84 ± 0.53	3.98 ± 0.51	3.67 ± 0.53	<0.0001**
Prior Pacemaker		28 (11.6)	10 (7.5)	18 (16.7)	0.022*
Prior CABG Surgery		38 (15.7)	19 (14.2)	19 (17.6)	0.291
Prior Aortic Valve Surgery		32 (13.2)	14 (10.4)	18 (16.7)	0.110
TAVR Status Elective		236 (97.5)	133 (99.3)	103 (95.4)	0.064
TAVR Operative Risk Assessment					<0.0001**
	Low	66 (27.3)	50 (37.3)	16 (14.8)	-
	Intermediate	99 (40.9)	53 (39.6)	46 (42.6)	-
	High	74 (30.6)	30 (22.4)	44 (40.7)	-
	Inoperable	3 (1.2)	1 (0.7)	2 (1.9)	-
STS Risk Score (mean ± SD)		4.6 ± 4.2	3.9 ± 4.1	5.4 ± 4.1	<0.0001**

Abbreviations: CABG, coronary artery bypass grafting; TAVR, transcatheter aortic valve replacement; SD, standard deviation; STS, society of thoracic surgeons; CPS, cerebral protection system. Data are presented as n (%), unless otherwise indicated. *p < 0.05, **p < 0.01.

Multivariate analysis of one-year endpoints in the 242 consecutive patients was done using Cox Proportional Hazards Regression modeling, including as covariates the propensity score and all factors that were significantly different between CPS and non-CPS patients at baseline. We did perform a power calculation to determine the effect size difference that our analyses would be sensitive in detecting given our sample size (N = 242), at a power level of 0.8 and *p*-value of <0.05. These calculations indicated that our sample size would have been able to detect a difference in the composite endpoint of 12% between the 2 groups. A value of *p* < 0.05 was used to determine the statistical significance of all tests. Analyses were performed using the IBM/SPSS statistical software package version 28.0.1 (IBM Corporation, Amonk, NY, USA). A sensitivity analysis was performed to determine the effect of the propensity score in controlling for the group differences in the Cox Proportional Hazard analysis of late clinical events, including the CPS group and propensity score alone in a model, and then performing another model with the CPS groups and only the baseline factors that were used to develop the propensity score.

## 3. Results

### 3.1 Patient Characteristics

Demographic and baseline clinical factors are presented in Table 1. Sentinel CPS patients were significantly more likely to be male, and less likely to be on dialysis, have a history of CVA, on immunosuppressant therapy, and have chronic lung disease. There was no significant difference in the prevalence of bicuspid aortic valves between groups (*p* = 0.218). Pre-TAVR procedural characteristics and patient status are displayed in Table 2. Periprocedural, Sentinel CPS patients had significantly higher hemoglobin and total albumin levels, lower creatinine, and were less likely to have a pacemaker or classified as high risk by the TAVR operator. Both STS Risk score and TAVR operative risk assessment were significantly less in the Sentinel CPS group (*p <* 0.0001).

### 3.2 Procedural Details

Of the 242 patients, 129 (53.3%) received Edwards Valves (Sapien 3 or S3 Ultra), while 113 patients received Medtronic CoreValves (Evolut, Evolute Pro, or Evolut Pro Plus). The preferred approach was transfemoral (95.5%), followed by subclavian (3.3%). A total of 18 patients (7.4%) underwent valve-in-valve (ViV) procedures with no significant differences between groups (*p* = 0.633) (**Supplementary Table 1**). Of the 134 Sentinel CPS patients, there were no perioperative complications with device deployment, removal or access site.

### 3.3 In-Hospital/30-Day Outcomes 

The occurrence of perioperative stroke post-TAVR was not significantly different between CPS and non-CPS patients (0.0% vs. 1.9%; *p* = 0.198), nor were there any differences in mortality (0.75% vs. 1.9%; *p* = 0.419). Sentinel CPS patients had slightly longer fluoroscopic exposure time (20.2 ± 8 vs. 17.7 ± 8 mins), lower post-procedure creatinine (1.01 ± 0.61 vs. 1.32 ± 1.10), received fewer red blood cell transfusions (5.2% vs. 13.0%), and had shorter length of stays (2.5 ± 2.2 vs. 3.3 ± 3.2 days). None of these comparisons were statistically significant after controlling for STS Risk score. A total of 239 (98.8%) patients were successfully discharged following TAVR, with three in-hospital mortalities (1 cardiac, 1 vascular, and 1 neurologic death).

### 3.4 One-Year Outcomes

The average time to latest follow-up was 12.2 months (12.2 ± 3.2) for the Sentinel CPS group and 11.9 months (11.9 ± 3.7) for the no Sentinel CPS patients (*p* = 0.539). Follow-up was 97% complete, with only 8 patients lost to 12-month follow-up. Echocardiographic follow-up was 100% complete. Unadjusted analyses showed a lower occurrence of the composite endpoint in CPS patients at one-year when compared to non-CPS patients (8.3% vs. 17.0%; *p* = 0.034).

To adjust for baseline group differences, a propensity score was developed using all factors listed in Tables 1,2. The overall propensity score was 0.548 and the score for CPS patients was 0.675 and 0.394 for non-CPS patients (*p* < 0.0001). Cox (proportional hazards) regression analysis was used to evaluate for the occurrence of the composite endpoint at one-year, with the propensity score and all significantly different baseline group factors used as covariates in the models. Adjusted analyses demonstrated Sentinel CPS was not significantly associated with the composite endpoint (Adj HR = 0.609, 95% CI 0.244–1.523; *p* = 0.289). In the Cox model of the composite endpoint, STS Risk score, current dialysis, and immunosuppressant therapy use were all significantly associated with the endpoint (Table 3). Unadjusted analyses of stroke showed no difference in the incidence of stroke at one-year (0.8% vs. 2.8%; *p* = 0.233), nor did adjusted analyses (Adj HR = 0.149, 95% CI 0.013–1.763; *p* = 0.132).

**Table 3.** **COX Proportional Hazards Regression Models for the composite endpoint at 12 months follow-up with adjusted hazard ratios and 95% confidence intervals comparing Sentinel CPS vs. no Sentinel CPS**.

	Adj HR	95% CI	*p* value
STS Risk Score	1.150	1.068–1.239	0.001**
Current dialysis	13.947	1.414–137.61	0.024*
Immunosuppressant Therapy	12.333	2.667–56.810	0.001**
Sentinel Devise Use	0.609	0.2441–1.523	0.289
Propensity Score	19.037	1.606–225.64	0.020*

Abbreviations: CI, confidence interval; CPS, cerebral protection system; HR, hazard ratio; STS, society of thoracic surgeons; CVA, cerebrovascular accidents. Composite endpoint = death, CVA, bleeding, and vascular event. This model includes the propensity score and all statistically significant factors from Tables 1,2. Only factors that were significant in the model plus Sentinel use are reported here. *p < 0.05, **p < 0.01.

### 3.5 Sensitivity Analysis

Sensitivity analyses were performed using several Cox models to assess the effect of the propensity score. In the first Cox model with composite clinical events as the outcome, the CPS group was placed in the model, and this produced an adjusted hazard ratio of 0.463 (95% CI 0.219–0.929, *p* = 0.045). A *second Cox model* was done that then included the CPS group and the updated propensity score. The CPS group had an Adj HR of 0.566 (95% CI, 0.244–1.322, *p* = 0.189) and the propensity score had an Adj HR of 0.489 (95% CI 0.104–2.291, *p* = 0.364). In the *third Cox model*, the propensity score was left out and all of the factors that had been used to construct the updated propensity score were included in the model with the CPS group. In this model, the CPS group had an Adj HR of 0.436 (95% CI 0.157–1.211, *p* = 0.111). Other significant factors included the STS Risk score (Adj HR = 1.263, *p* < 0.001), dialysis (Adj HR = 0.294, *p *= 0.037) and immunosuppressant therapy (Adj HR = 5.367, *p* = 0.036). These analyses demonstrated that the updated propensity score was successful in adjusting for the differences between the CPS groups, since this variable did lose significance in that model, and a similar effect was seen on the CPS group when all of the baseline variables were used in place of the updated propensity score in the final model.

## 4. Discussion

This is a real-world study, performed at a single center on a consecutive series of patients undergoing TAVR. With no existing guidelines on CPS device use and patient selection in TAVR, the main objective of our study was to compare the characteristics of patients who tend to receive Sentinel CPS devices with those who do not in a “real-world” setting. We also examined the occurrence of post-TAVR complications between groups, namely in-hospital and one-year stroke. In this study, we found patients who received a Sentinel CPS device were more likely to be healthier and deemed less complex when compared to concurrent patients who did not receive a device. In addition, we demonstrated no statistically significant association between device use and decreased in-hospital/30-day post-TAVR stroke or in the occurrence of our composite endpoint (death, stroke, and bleeding/vascular events) at one-year.

With RCT demonstrating similar [27] or superior [28] clinical outcomes of TAVR to SAVR in low surgical risk patients, the extension of TAVR to young and low-operative risk patients has rapidly grown [29, 30]. These findings highlight the necessity to ensure TAVR remains safe, with a low risk of adverse clinical events, especially in a younger population with longer life expectancies. Specifically, CVA post-TAVR is a devastating complication and remains non-negligible ranging up to 5.5% at 30-days and 8.2% at 1 year [3, 8, 9, 31]. Major CVA post-TAVR is a known independent predictor of morbidity and mortality [15, 32, 33], with moderate to severe permanent disability leading to dependency in up to 40% of survivors and a further 80% facing social isolation and significant financial stress [29, 34, 35]. These patients represent a significant societal healthcare burden and contribute to increased financial strain on healthcare systems.

More than half of post-TAVR CVA are due to procedure-related emboli [29], which led to the development of embolic protection devices like the Sentinel CPS. To date, clinical studies have not conclusively determined the efficacy of these devices in preventing CVA [29, 36, 37]. The Sentinel CPS is the most studied embolic protection device in TAVR, and the most commonly used device worldwide [32]. Reviewing results from the three largest RCTs on this device totaling 528 patients (MISTRAL-C, CLEAN-TAVI, and SENTINEL), none were able to demonstrate a reduction in the clinical endpoint of mortality and/or stroke [15, 16, 18]. Lacking statistical power secondary to small sample sizes and a low incidence of post-TAVR CVA, none of these trials were able to use CVA as a primary endpoint [29, 32, 38]. Several meta- and propensity matched-analyses have also tried to examine Sentinel CPS efficacy [29], with available meta-analyses yielding similar results (due to significant study overlap) and failing to demonstrate device efficacy [14, 39, 40]. Propensity matched analyses using data from multiple large registries have also been unable to consistently demonstrate significant reductions in stroke with Sentinel CPS use [36, 37, 41, 42].

Although our study was small and not randomized, our findings are consistent with current literature on the lack of Sentinel CPS efficacy on stroke reduction, and representative of real-world TAVR data. A total of 2 in-hospital (0.8%) and 4 one-year (1.7%) CVA occurred post-TAVR in our study. Despite a lower numerical incidence of CVA in the Sentinel CPS group at both time points, unadjusted analyses demonstrated no statistically significant difference between groups (0 vs. 2; *p* = 0.198 and 1 vs. 3; *p* = 0.233, respectively). To account for baseline differences and non-randomization, a propensity score was developed using all factors in Table 1 and Table 2, which was then used in multivariate analysis. Even after adjustment, the adjusted hazard ratio for the CPS group still showed no difference in stroke incidence at 1 year (*p* = 0.132), with no variables included in the analysis reaching statistical significance. Like other studies though, our small sample size and low incidence of CVA, reduced our statistical power and ability to demonstrate any reduction in stroke. Our overall one-year stroke rates (1.7%) were even lower than 30-day stroke rates (>2.3%) reported by large TAVR registries like the TVT [6, 35].

While unadjusted analysis of our composite endpoint demonstrated a significantly lower occurrence in the CPS group (*p *= 0.034), after adjustment this was no longer true (*p* = 0.289, Table 3). Interestingly, being on immunosuppressant therapy and dialysis were both statistically significant major risk factors of the composite endpoint in TAVR patients (Table 3). This is not surprising, considering our composite endpoint included death and stroke. Overall mortality in dialysis patients is 10–20 times higher than in the general population, with a one-year survival rate of 30% in adults >65, almost doubling to 54% in patients >85 [43]. Furthermore, chronic kidney disease and dialysis are both known risk factors for cerebrovascular disease, and are associated with higher stroke rates, worse stroke severity, outcomes, and mortality [44]. Similarly, patients on immunosuppressant therapy have increased mortality rates [45], secondary to increased rates of infection [46] and malignancy [47, 48]. While low BMI (<20 ${\mathrm{kg/m}}^{2}$) has been shown to be an independent predictor of increased short and long-term mortality in real-world patients undergoing TAVR [49], this finding was not demonstrated in our study as the mean BMI of all patients in our study was 29.2 ± 7 with no statistically significant difference in distribution between groups.

With no benefit in stroke reduction or the occurrence of our composite endpoint demonstrated with Sentinel CPS use, we focused on exploring why some patients were selected to receive the device while some were not. Comparing baseline and pre-TAVR procedural characteristics/patient status between groups, patients receiving the Sentinel CPS device were overall healthier at baseline and deemed less complex (Tables 1,2). No Sentinel CPS patients had significantly higher incidences of dialysis (*p* = 0.016), chronic lung disease (*p* = 0.012), prior TIA (*p* = 0.020) and pacemaker (*p* = 0.022), and were more likely to be on immunosuppressant therapies (*p* < 0.001) when compared to the Sentinel CPS group. Pre-TAVR, no Sentinel CPS patients also had lower hemoglobin (*p* = 0.001) and albumin (*p* < 0.0001) levels – both potent markers of frailty and mortality following TAVR [50], as well as higher creatinine levels (*p* = 0.016). Examining patient complexity, patients receiving Sentinel CPS also had significantly lower STS Risk Scores (*p* < 0.0001) and TAVR operative risk assessments (*p* < 0.0001).

With no guidelines available to aid decision making on CPS device use and patient selection, the data presented here suggest that at our institution, Sentinel CPS was used more frequently in men (*p* = 0.006) and patients who were healthier and less complex. These findings are not surprising, since as patient complexity increases, patients tend to develop more contraindications or relative contraindications to Sentinel CPS use. For example, patients with COPD may have had multiple arterial punctures for blood gases in the past, and thus have a difficult radial artery to access for device use. Alternatively, patients on dialysis or those with a history of prior TIA may be more likely to have significant carotid disease or aortic arch and great vessel calcification, precluding the use/deployment of the Sentinel CPS. Finally, men typically have larger radial arteries and less tortuosity, facilitating the deployment of Sentinel CPS when compared to women.

While the final decision rests with the operator, during the time period of this study our Valve team’s policy was to consider Sentinel CPS in all patients. Investigating the possible driving factors behind patient selection in our study revealed those receiving a device were likely chosen simply based on the ease and safety of device placement. Operators were more likely to deploy the Sentinel CPS in healthier patients, who inherently have lower baseline stroke risks, and were least likely to have derived any benefit from device placement. While the mechanism of benefit for Sentinel CPS has very strong theoretical plausibility, recent randomized data is consistent with our findings, suggesting the net effect on objective clinical endpoints such as stroke is non-significant [15, 51]. In the PROTECTED TAVR study, a multi-center, multi-country RCT comparing the effects of Sentinel use on the incidence of perioperative stroke reduction totaling 3000 patients, Kapadia *et al*. [51] also demonstrated no differences in stroke (Sentinel 2.3% vs. No Sentinel 2.9%, *p* = 0.30) or mortality (0.5% vs. 0.3%) at 72 hours or before discharge.

With accumulating evidence against device benefit, our institution has moved towards a “do no harm” approach first when selecting patients for device use. If a patient has anatomic or clinical factors that make device use a risk for complication, no device is used or even attempted. Instances where we recommend against Sentinel CPS use include: (1) Patients who have weakly palpable or occluded radial arteries that preclude arterial access; (2) Severe tortuosity in the subclavian precluding the ability to adequately deploy the device; (3) Patients with severe atherosclerosis or calcification in the brachiocephalic or left common carotid arteries, where manipulations in this area may cause greater risk than benefit; and (4) patients for whom the right radial is used for the pigtail catheter or a guide catheter for coronary protection due to inadequate femoral access.

In summary, with no definitive answer on device effectiveness on stroke reduction, or clear evidence of clinical benefit [29, 51] to date, the utility of Sentinel CPS use in TAVR remains questionable. Associated with substantial morbidity, longer hospital stays, increased mortality, readmission rates, and hospital costs [3, 6, 7, 8, 9, 52, 53], CVA post-TAVR are devastating complications and adversely affect the healthcare system. Despite these implications, it is unlikely Sentinel CPS use will become a clinical standard based on current evidence. With an already low occurrence of stroke post-TAVR, and significant device-associated costs (~$3000 USD) that are not recuperated and compounded by a high number needed to treat (NNT: 125 to prevent 1 disabling stroke) [51], Sentinel CPS use may not be the answer to lowering stroke incidence in TAVR. Until further evidence supporting efficacy in stroke reductions becomes available, device use will likely remain operator and patient anatomy dependent, with some consideration to patient request and/or institutional budgets.

## 5. Limitations

While our study was not randomized for patient assignment to Sentinel, several statistical control methods were used to allow comparison between groups. However, other potentially significant unmeasured confounders may still exist. Patient frailty represents one such confounder, as no formal measure of frailty was available in our study, and may have been an important unaccounted difference between groups. While our sample size was small, and therefore our results not adequately powered to compare event rates between patients in the Sentinel CPS group with those who did not receive one, our results do represent a consecutive series in a real-world clinical setting with control of the operators performing TAVR. Lastly, no routine neurologist assessment or neuroimaging surveillance was performed in our study to evaluate for CVA post-TAVR. Although this likely resulted in a lower perioperative CVA detection rate, it is unlikely to have significantly affected results of our study endpoints, especially considering the already exceedingly low occurrence of stroke (1.7%). Furthermore, all strokes reported in our study were diagnosed (and verified) using the recognized standardized VARC-3 definition criteria for stroke, eliminating clinical outcome definition variability.

## 6. Conclusions

In conclusion, our study demonstrates that the Sentinel CPS device is more likely to be used in patients who are less sick and complex compared with other TAVR patients. This is likely a consequence of healthier patients having safer and more amenable anatomy for device use. Device use was not significantly associated with a lower occurrence of perioperative stroke or reduction in the composite endpoint (death, stroke, bleeding, and vascular events) at one-year following TAVR. Further research involving randomization and larger multicenter studies is required to understand how CPS is utilized in the “real-world” and to identify its potential role in reducing stroke after TAVR.

## Data Availability

The datasets generated and/or analyzed during the current study are not publicly available due to privacy/ethical restrictions, but are available from the corresponding author on reasonable request.

## Supplementary Material

Supplementary material associated with this article can be found, in the online version, at https://doi.org/10.31083/j.rcm2501003.
