# Supplementary material for: Patient Characteristics and Outcomes Associated with Sentinel Protection Device Use in Patients with Aortic Valve Disease Undergoing TAVR in a “Real-World” Setting
Source: Rev Cardiovasc Med. 2024 Jan 4;25(1):3. doi: 10.31083/j.rcm2501003 (PMC11262339; doi:10.31083/j.rcm2501003)
Supplement: Supplementary file 1 [file 2153-8174-25-1-003-s1.docx]

***Supplementary Table 1. TAVR Devices and Access Routes***

|  | **Sentinel CPS**  **N=132 (55.5%)** | **No Sentinel CPS**  **N=106 (45.5%)** | **Total** |
| --- | --- | --- | --- |
| **Valve Type*** | | |  |
| **Edwards**  Sapien 3  Sapien 3 Ultra  **Medtronic**  CoreValve Evolut  CoreValve Evolut Pro  CoreValve Evolut Pro Plus | 67 (50)  9 (6.7)  6 (4.5)  13 (9.7)  39 (29.1) | 50 (42.7)  3 (2.8)  16 (14.8)  21 (19.4)  18 (6.7) | 117 (48.3)  12 (5)  22 (9.1)  34 (14)  57 (23.6) |
| **Access Route** | | |  |
| Femoral | 132 (98.5) | 99 (91.7) | 231 (95.5) |
| Subclavian | 2 (1.5) | 6 (5.6) | 8 (3.3) |
| Axillary | 0 (0) | 1 (0.9) | 1 (0.4) |
| Transaortic | 0 (0) | 1 (0.9) | 1 (0.4) |
| Transapical | 0 (0) | 1 (0.9) | 1 (0.4) |
| **Valve in Valve (ViV)** | | |  |
| No ViV | 125 (93.3) | 99 (91.7) | 224 ((92.6) |
| ViV | 9 (6.7) | 9 (8.3) | 18 (7.4) |

* Valve sizes ranged between 23 mm and 34 mm.
